# SOX9 is a key component of RUNX2-regulated transcriptional circuitry in osteosarcoma

**DOI:** 10.1186/s13578-023-01088-2

**Published:** 2023-07-25

**Authors:** Young-Im Kim, Yu-Chou Tseng, Gamze Ayaz, Shasha Wang, Hualong Yan, Wendy du Bois, Howard Yang, Tao Zhen, Maxwell P. Lee, Paul Liu, Rosandra N. Kaplan, Jing Huang

**Affiliations:** 1 Cancer and Stem Cell Epigenetics Group, Laboratory of Cancer Biology and Genetics, Center for Cancer Research, National Cancer Institute, Bethesda, MD USA; 2 High-Dimension Data Analysis Group, Laboratory of Cancer Biology and Genetics, Center for Cancer Research, National Cancer Institute, Bethesda, MD USA; 3 Translational and Functional Genomics Branch, National Human Genome Research Institute, Bethesda, MD USA; 4 Tumor Microenvironment Section, Pediatric Oncology Branch, Laboratory of Cancer Biology and Genetics, Center for Cancer Research, National Cancer Institute, Bethesda, MD USA

**Keywords:** Epigenetics, JMJD1C, Osteosarcoma, RUNX2, SOX9, Transcription

## Abstract

**Background:**

The absence of prominent, actionable genetic alternations in osteosarcomas (OS) implies that transcriptional and epigenetic mechanisms significantly contribute to the progression of this life-threatening form of cancer. Therefore, the identification of potential transcriptional events that promote the survival of OS cells could be key in devising targeted therapeutic approaches for OS. We have previously shown that RUNX2 is a transcription factor (TF) essential for OS cell survival. Unfortunately, the transcriptional network or circuitry regulated by RUNX2 in OS cells is still largely unknown.

**Methods:**

The TFs that are in the RUNX2 transcriptional circuitry were identified by analyzing RNAseq and ChIPseq datasets of RUNX2. To evaluate the effect of SOX9 knockdown on the survival of osteosarcoma cells in vitro, we employed cleaved caspase-3 immunoblotting and propidium iodide staining techniques. The impact of SOX9 and JMJD1C depletion on OS tumor growth was examined in vivo using xenografts and immunohistochemistry. Downstream targets of SOX9 were identified and dissected using RNAseq, pathway analysis, and gene set enrichment analysis. Furthermore, the interactome of SOX9 was identified using BioID and validated by PLA.

**Result:**

Our findings demonstrate that SOX9 is a critical TF that is induced by RUNX2. Both in vitro and in vivo experiments revealed that SOX9 plays a pivotal role in the survival of OS. RNAseq analysis revealed that SOX9 activates the transcription of *MYC*, a downstream target of RUNX2. Mechanistically, our results suggest a transcriptional network involving SOX9, RUNX2, and MYC, with SOX9 binding to RUNX2. Moreover, we discovered that JMJD1C, a chromatin factor, is a novel binding partner of SOX9, and depletion of JMJD1C impairs OS tumor growth.

**Conclusion:**

The findings of this study represent a significant advancement in our understanding of the transcriptional network present in OS cells, providing valuable insights that may contribute to the development of targeted therapies for OS.

**Supplementary Information:**

The online version contains supplementary material available at 10.1186/s13578-023-01088-2.

## Introduction

Osteosarcoma (OS) is the most frequently occurring type of bone cancer among children and adolescents. While the 5-year survival rate for OS is greater than 70%, this drops to just 25% for those who have recurrent or metastatic tumors. In addition, the conventional treatment for osteosarcoma has remained unchanged for more than 30 years and frequently causes significant adverse effects. To date, no targeted or immunotherapies for OS have been approved by the FDA [1]. Therefore, it is crucial to develop innovative therapeutic approaches for osteosarcoma that minimize the adverse effects of chemotherapy and improve patients’ quality of life. However, genome-wide sequencing studies of human OS tumors have failed to identify any significant, actionable oncogenic mutations or genetic alterations, with the exception of frequent losses of *TP53* and *RB1* genes [2, 3]. A plausible explanation for this observation is that OS biology may be influenced by transcriptional or epigenetic mechanisms that are beyond the scope of exome sequencing detection.

The RUNX2 (RUNX family transcription factor 2) protein is a crucial transcription factor (TF) for the survival of OS cells. In normal development, RUNX2 plays a role in regulating the maturation of osteoblasts. [4]. In OS tumors, the RUNX2 gene is frequently amplified [5]. The majority of OS cell lines exhibit elevated levels of RUNX2 expression, which in turn triggers *MYC* transcription and promotes the survival of OS cells. [6]. A recent study revealed that RUNX3, another member of the RUNX family, plays a significant role in osteosarcoma development by inducing *MYC* expression [7]. Because core TFs for a particular cancer type tend to form an interconnected circuitry or network [8], it is crucial to identify other TFs within the core circuitry of OS that interact with RUNX2 and RUNX3 to promote cell survival.

SOX9 is a member of the HMG-box class of DNA-binding TFs and is instrumental in chondroblast maturation [9]. Specifically within chondrocytes, SOX9 regulates the expression of genes involved in cell cycle progression and differentiation [10]. In the context of OS, SOX9 acts as a mediator for FOS-induced chondroblastic OS and contributes to OS tumorigenesis [11, 12]. However, its precise roles in the survival of OS cells and the underlying transcriptional circuitry remain poorly understood.

In this study, we aimed to gain a more comprehensive understanding of the transcriptional network governed by RUNX2 in OS cells, which led to the discovery of SOX9 as a downstream target of RUNX2. Mechanistic studies demonstrated that SOX9 interacts with RUNX2 and is an integral component of the RUNX2-regulated transcriptional circuitry that promotes OS cell survival. Furthermore, SOX9 collaborates with RUNX2 to activate MYC expression. Additionally, we have identified a novel binding partner of SOX9, JMJD1C, which may represent a potential therapeutic target for targeted therapy in OS.

## Result

### *SOX9* is a direct target of RUNX2

**Fig. 1:**
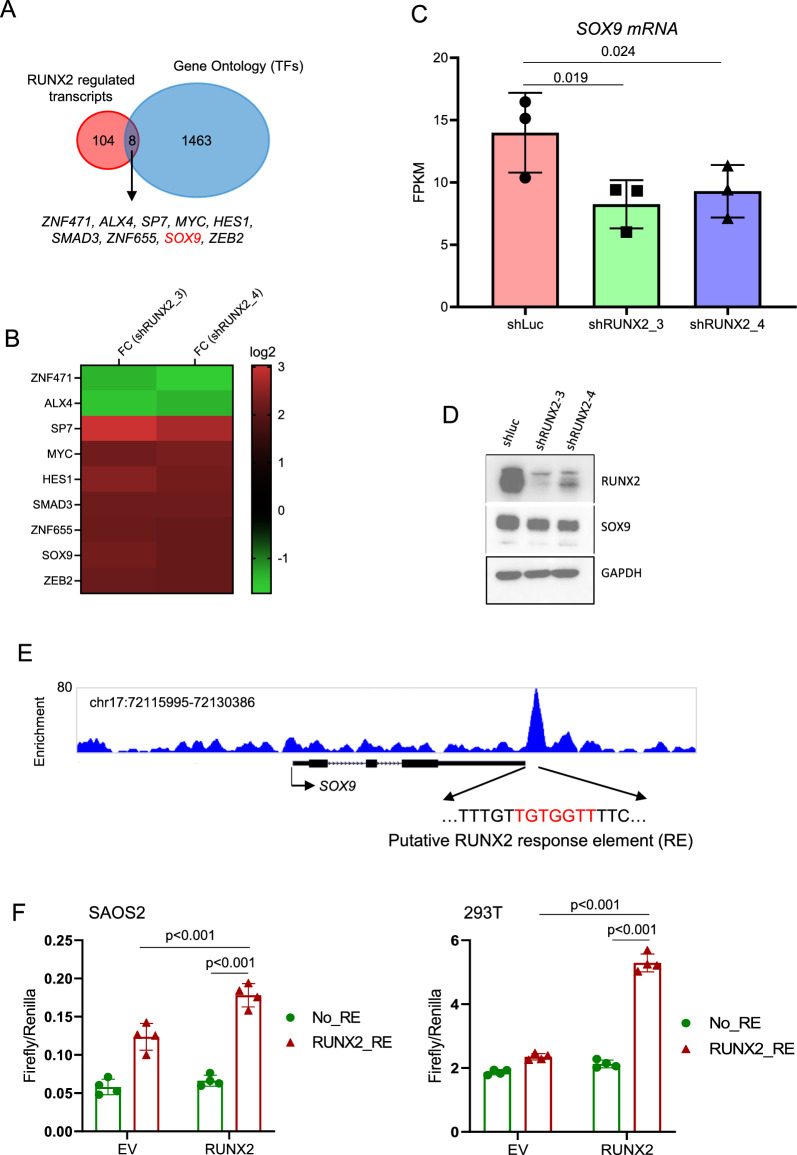
SOX9 is a direct target of RUNX2 in OS. **A**, Venn diagram showing the strategy of identifying transcription factors regulated by RUNX2. RUNX2-regulated transcripts were derived from public datasets (GSE76937 and GSE77352). The GO term for transcription factors is 0003700. **B**, Heatmap showing the fold change (FC) of shLuc versus shRUNX2_3 or shRUNX2_4 for the eight transcription factors regulated by RUNX2. **C**, RNAseq showing FPKM (fragment per kilobase per million reads) of SOX9. The RNAseq data is from a public dataset, GSE77352. **D**, Immunoblotting showing the effect of RUNX2 knockdown of SOX9 in SAOS2 cells. **E**, ChIPseq showing the binding of RUNX2 on the *SOX9* locus in SAOS2 cells. The ChIPseq data is from a public dataset, GSE76937. The putative RUNX2 response element (RE) was shown in red, and the DNA sequence comprising this RE was cloned to a reporter, pGL4.23 (See Methods for details). **F**, Reporter assays in SAOS2 (left) and 293 T (right) cells showing that the putative RUNX2 response element (RUNX2_RE) is involved in the regulation of *SOX9* by RUNX2. No_RE: empty pGL4.23 vector (No response element); EV: empty vector (without RUNX2 overexpression). n = 4, p values are from the t-test

We previously showed that RUNX2 is a TF required for the survival of OS cells [6]. To identify other TFs in this network, we overlapped RUNX2-regulated genes (Additional file 1: Table S1) with those encoded DNA sequencing-specific TFs (Gene Ontology, GO: 0003700). This analysis revealed nine TFs that were downstream genes of RUNX2. These TFs are *ZNF471*, *ALX4*, *SP7*, *MYC*, *HES1*, *SMAD3*, *ZNF655*, *SOX9*, and *ZEB2* (Fig. 1A). RUNX2 represses *ZNF471* and *ALX4* while activating the others (Fig. 1B). Among them, MYC has previously been shown as a crucial survival TF in OS [6]. SP7 is a critical factor for osteoblast maturation and has been shown to have anti-tumor activity in murine OS [13, 14]. HES1 is an important mediator of the NOTCH signaling pathway, and SMAD3 mediates the Transforming growth factor-β (TGF-β) signaling pathway. Both pathways are involved in OS progression [15, 16]. ZEB2 is involved in epithelial-mesenchymal transition (EMT) and may play a role in OS metastasis[17]. The roles of ZNF471, ALX4, and ZNF655 in OS are unknown. Our focus on SOX9 is based on our previous study, which demonstrated that SOX9 promotes the development of chondroblastic osteosarcoma from mesenchymal stem/stromal cells [12]. However, it is currently unclear whether the survival of osteosarcoma cells is dependent on SOX9. RUNX2 knockdown in SAOS2 reduced *SOX9* mRNA and protein levels (Fig. 1C, D), and the regulation of SOX9 by RUNX2 is conserved in mouse osteosarcoma cells (Additional file 2: Fig. S1). We observed that the decrease in *SOX9* expression was only mild upon RUNX2 knockdown (Fig. 1D), which indicates the involvement of other factors, potentially including RUNX3. ChIPseq showed that RUNX2 binds to the downstream region of the *SOX9* locus (Fig. 1E), suggesting that *SOX9* is a direct target of RUNX2. To test whether the binding site of RUNX2 is involved in the regulation of *SOX9*, we cloned the DNA fragment containing the putative RUNX2 response element (RE) (Additional file 2: Figure S2) into a reporter and performed luciferase assay. The results showed that this RUNX2 RE regulates *SOX9* expression (Fig. 1F).

### SOX9 is required for the survival of OS cells in vitro

**Fig. 2:**
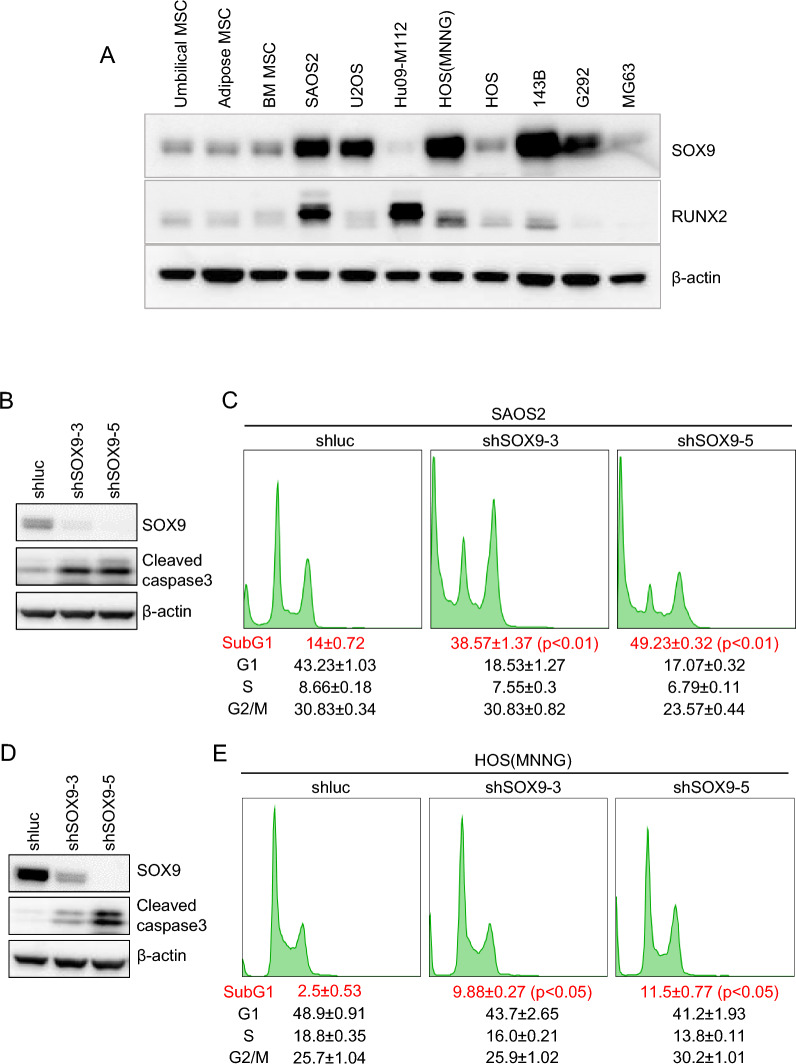
SOX9 is a survival factor of OS cells in vitro. **A**, Immunoblotting showing the protein levels of SOX9 and RUNX2 across different OS cell lines and mesenchymal stem/stromal cells (MSCs). **B** and **D**, Immunoblotting showing SOX9, cleaved caspase 3, and β-actin levels in SAOS2 (**B**) and HOS-MNNG (**D**) cells 6 days after SOX9 knocdown. **C** and** E**, Propidium iodide staining showing the effect of SOX9 knockdown on cell cycle and apoptosis (sub-G1) in SAOS2 (**C**) and HOS-MNNG (**E**) cells 6 days after the knockdown

We first examined the expression levels of SOX9 and RUNX2 across different OS cell lines. SOX9 levels were high in SAOS2, U2OS, HOS(MNNG), 143B, and G292 cells (Fig. 2A). Interestingly, we did not find a significant correlation between the levels of SOX9 and RUNX2 proteins. This lack of correlation could be attributed to the limitation of our study that we only investigated the regulation of SOX9 by RUNX2. Other TFs, such as RUNX1 or 3, PITX1, and LEF1, may also modulate the expression of SOX9 in OS cells [18, 19], and their activities may differ in these cell lines. Moreover, the overall protein level of SOX9, as assessed through immunoblotting, is influenced by multiple factors, including transcription, splicing, and translation processes. These regulatory steps may operate differently in various cell lines, leading to variations in the steady-state protein levels of SOX9.

To examine the role of SOX9 in OS cell survival and proliferation, we knocked down SOX9 in SAOS2 and HOS(MNNG) cells using short hairpin RNA (shRNA). In both cell lines, knockdown of SOX9 induced apoptosis, judged by increased cleaved caspase 3 and sub-G1 in the propidium iodide staining (Fig. 2B–E). Notably, the degree of apoptosis increase correlates with the knockdown efficiency of SOX9 shRNAs, suggesting that the observed effect is on target. Overall, SOX9 is a survival TF for OS cells.

### Depletion of SOX9 reduces proliferation and increases apoptosis of OS in vivo

**Fig. 3:**
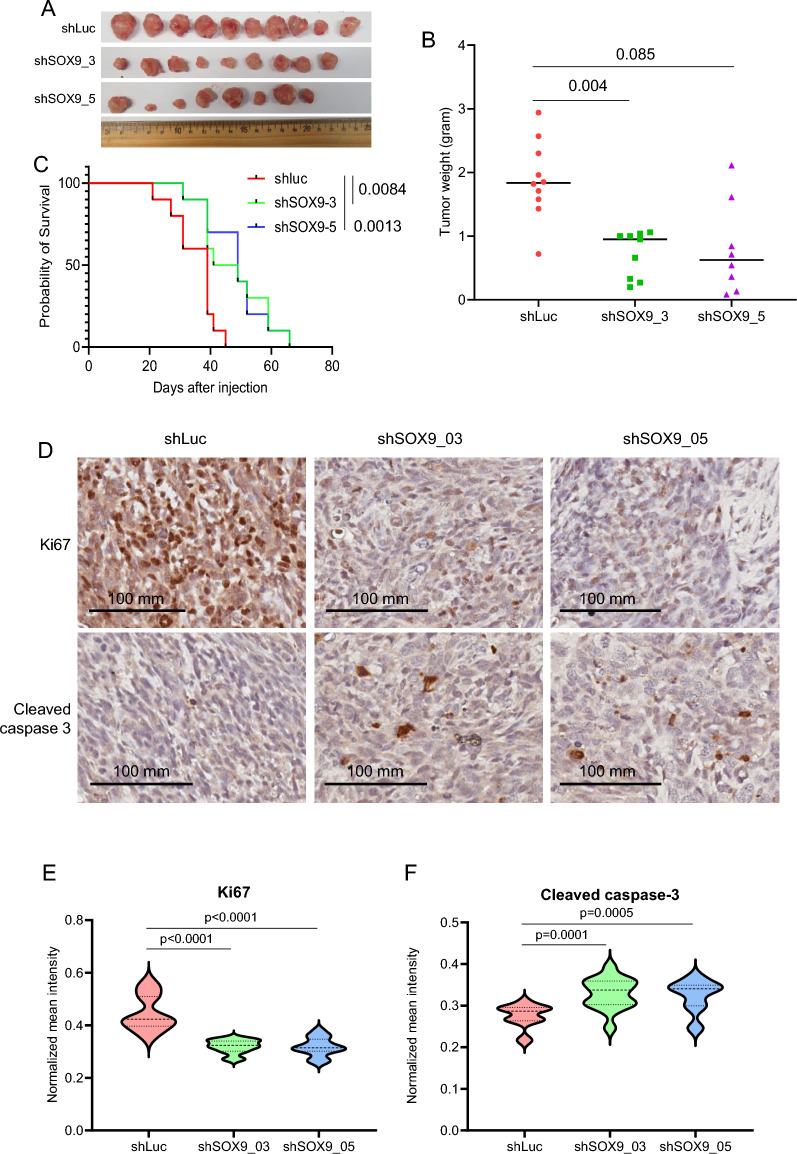
SOX9 is a survival factor of OS in vivo. **A**, Images of xenografts from SAOS2_shLuc (control), SAOS2_shSOX9_3 and SAOS2_shSOX9_5 taken at 63 days after cell injection. **B**, Tumor weights of xenografts from SAOS2 cells with different genotypes as indicated. n = 10 for shLuc; 9 for shSOX9_3 (one out of 10 mice had no tumor), and 8 for shSOX9_5 (two out of 10 mice had no tumor). p-values are from the Mann–Whitney test. **C**, Kaplan–Meier survival curve showing the overall survival of mice bearing subcutaneous tumors. Ten mice for each group. p-values are from the log-rank (Mantel-Cox) test. **D**, Representative images of immunohistochemistry (IHC) showing Ki-67 and cleaved caspase 3 staining. **E** and **F**, Quantitative analyses of Ki-67 (**E**) and cleave caspase 3 (**F**)

To test the effect of SOX9 depletion on OS tumor progression in vivo, we reduced the levels of SOX9 in SAOS2 cells using the two shRNAs and then transplanted cells into NOD-scid, IL2R gamma^null^ (NSG) mice. Knockdown of SOX9 reduced tumor growth (Fig. 3A, B) and prolonged the disease-free survival of the mice (Fig. 3C). It is interesting to note that one out of ten mice for shSOX9_3 and two out of ten mice for shSOX9_5 did not grow tumors. It is unknown whether the lack of tumors is due to SOX9 knockdown and/or other reasons. To further investigate the role of SOX9 in OS tumor growth in vivo, we performed immunohistochemistry (IHC) of Ki-67 (proliferative marker) and cleaved caspase 3 (apoptosis marker). SOX9 knockdown reduced Ki-67 staining signal and increased the cleaved caspase 3 signal (Fig. 3D–F). These in vivo data are consistent with those in vitro (Fig. 2C–E). Together, our results demonstrate that SOX9 is TF required for the survival of OS cells.

### SOX9-regulated genes and pathways in OS

**Fig. 4:**
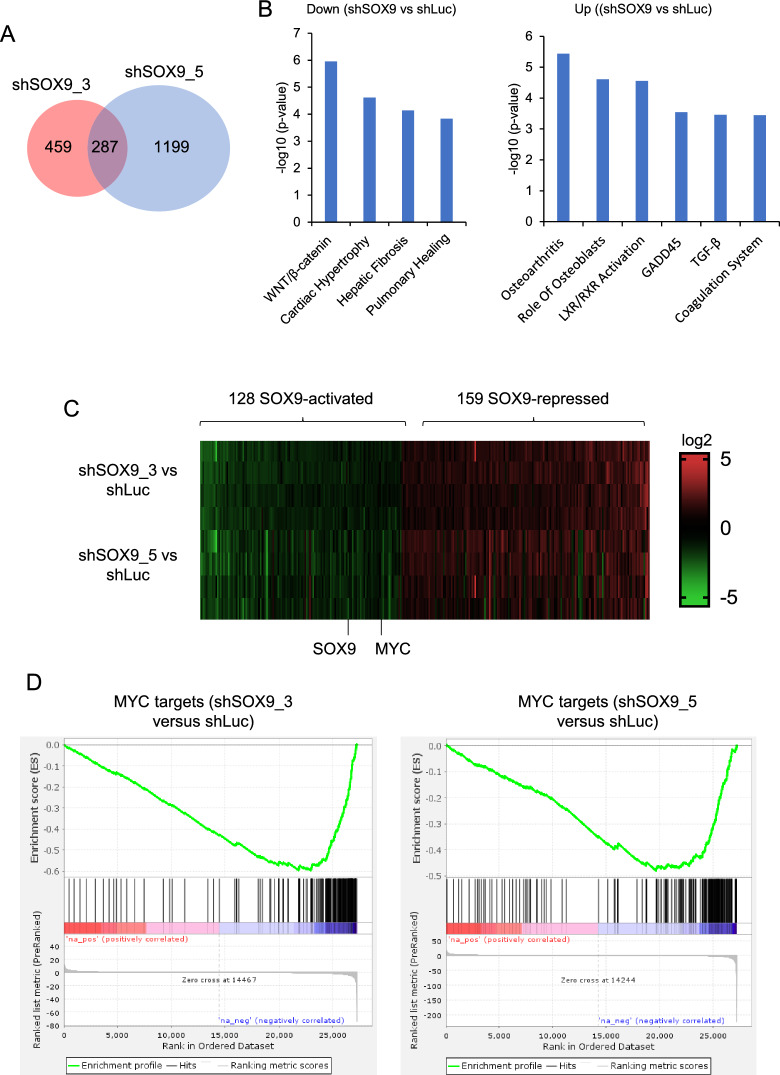
SOX9 regulates MYC expression. **A**, Venn diagram of SOX9-regulated transcripts by two shRNAs. **B**, Ingenuity Pathway Analysis (IPA) showing enriched pathways in SOX9-regulated transcripts (common between the two shRNAs). Left, pathways downregulated in shSOX9 (activated by SOX9); Right, pathways upregulated in shSOX9 (repressed by SOX9). **C**, Heatmap showing SOX9-activated and SOX9-repressed transcripts and highlighting *MYC* as a SOX9-activated gene. **D**, Gene Set Enrichment Analysis (GSEA) showing MYC gene signature is enriched in SOX9-regulated transcripts

We performed RNAseq analysis to investigate the molecular mechanisms underlying the pro-survival function of SOX9 in OS cells. Two hundred eighty-seven genes were regulated by both *SOX9* shRNAs (Fig. 4A and Additional file 3: Table S2). Ingenuity pathway analysis (IPA) revealed that the WNT/β-catenin, cardiac hypertrophy, hepatic fibrosis, and pulmonary healing pathways were significantly down-regulated in cells with SOX9 shRNAs, suggesting that SOX9 positively regulates these pathways (Fig. 4B). Pathways negatively regulated by SOX9 included osteoarthritis, the role of osteoblasts, LXR/RXR activation, GADD45, TGF-β, and the coagulation system. Among these 287 genes, 128 genes were activated by SOX9, while 159 were repressed (Fig. 4C). Expectedly, *SOX9* transcript levels were down in SOX9 shRNA samples. Interestingly, *MYC* is one of the SOX9-activated genes (Fig. 4C). Gene Set Enrichment Analysis (GSEA) showed that MYC-regulated genes were significantly enriched in SOX9-activated genes (Fig. 4D), suggesting that SOX9 may regulate the downstream genes of MYC through controlling MYC expression.

### SOX9 binds to RUNX2 and JMJD1C

**Fig. 5:**
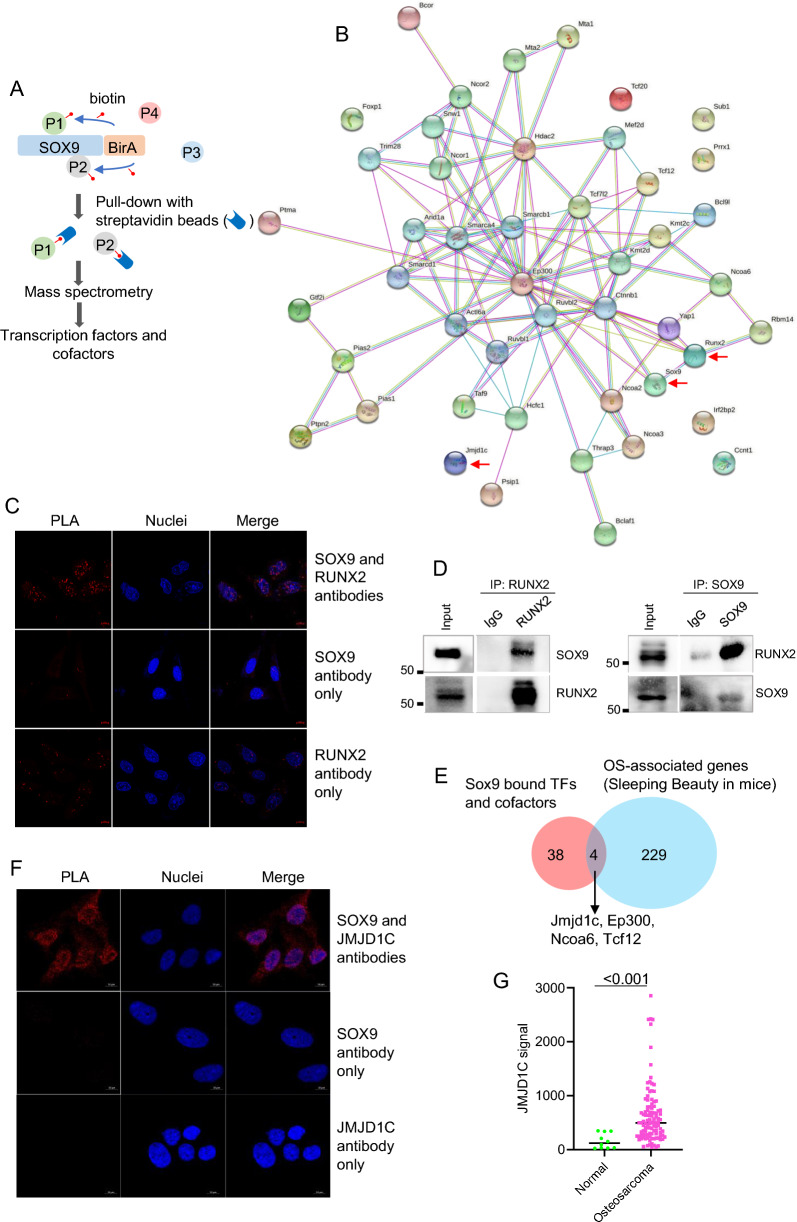
Proximitome analyses reveal RUNX2 and JMJD1C as novel binding partners of SOX9. **A**, Schematic showing BioID2 to identify the proximitome of SOX9 in DUNN cells. **B**, The network of SOX9 proximitome by STRING. **C**, Proximity ligation assay (PLA) showing the interaction between SOX9 and RUNX2. **D**, Co-immunoprecipitation of SOX9 and RUNX2 in SAOS2 cells. **E**, Venn diagram showing SOX9-interacting proteins that play a conservative role in mouse osteosarcomagenesis. OS-associated genes were from a public dataset [22]. **F**, PLA showing the interaction between SOX9 and JMJD1C. **G**, Analyses of public microarray datasets showing overexpression of JMJD1C in OS versus normal tissues

To further explore the SOX9 network in OS, we sought to identify the interacting partners of SOX9. To this end, we applied BioID assay to mouse OS DUNN cells [20]. SOX9 is conjugated to a promiscuous BirA enzyme, which biotinylates proteins in proximity to SOX9 (Fig. 5A). BioID assay identified 171 SOX9-interacting proteins (Additional file 4: Table S3). We then overlapped these 171 proteins with GO terms of transcription factors and cofactors (GO:0003700 and GO:0003712) to select those having transcriptional functions. This analysis resulted in 42 proteins (Additional file 5: Table S4), including SOX9 as a positive control. Using the STRING database [21], we examined the functional interactions between these 42 proteins (Fig. 5B). Interestingly, the STRING database suggested that SOX9 and RUNX2 potentially interact (Fig. 5B, arrows). To validate this finding in human SAOS2 cells, we utilized a proximity ligation assay (PLA), which is capable of detecting dynamic interactions under endogenous conditions. The PLA analysis revealed that SOX9 and RUNX2 are in close proximity in SAOS2 cells, suggesting an interaction between these two transcription factors (Fig. 5C). Indeed, we observed an interaction between SOX9 and RUNX2 using co-immunoprecipitation (Fig. 5D).

To identify other functionally important OS genes, we compared the 42 genes to those identified from a published screen using the Sleeping Beauty transposon system in mouse OS models [22]. The comparison revealed four genes, *JMJD1C*, *EP300*, *NCOA6*, and *TCF12* (*Jmjd1c*, *Ep300*, *Ncoa6*, and *Tcf12* in mouse, respectively), as potential driver genes in OS (Fig. 5E). *EP300* encodes p300, an enhancer acetyltransferase [23]. Functional interaction between SOX9 and p300 has been reported [24]. *NCOA6* is also known as *AIB3*, a commonly amplified gene in breast tumors [25]. However, its role in osteosarcoma has not been reported. The role of *TCF12* in osteosarcoma remains largely unknown. We focused on JMJD1C, a histone lysine demethylase, as histone lysine methylation and demethylation have recently emerged as important regulatory steps in cancer [26]. The PLA showed that SOX9 and JMJD1C interact in SAOS2 cells (Fig. 5F). An analysis of public microarray datasets showed that JMJD1C is overexpressed in human OS tumors compared to normal tissues (Fig. 5G), further suggesting that JMJD1C is critical for OS etiology.

### JMJD1C depletion reduces OS xenograft growth

**Fig. 6:**
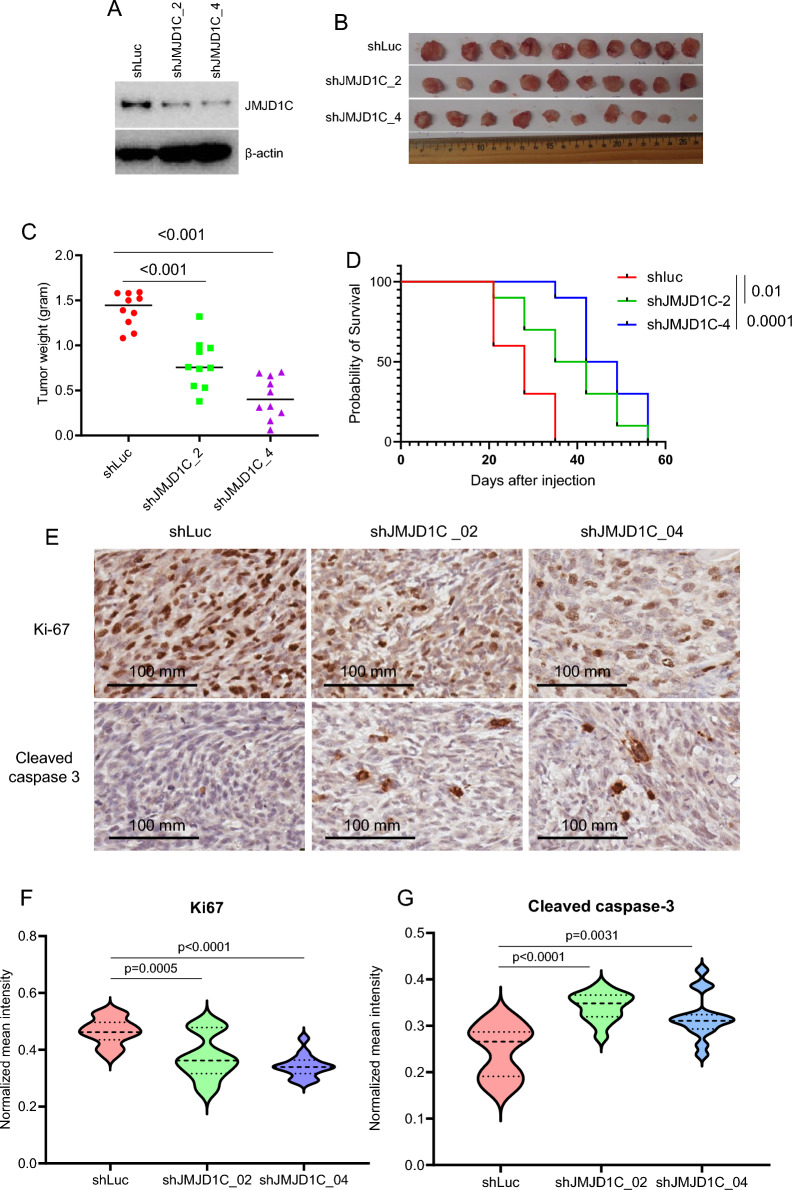
JMJD1C is required for the growth of OS in vivo. **A**, Immunoblotting showing knockdown of JMJD1C in SAOS2 cells. **B**, Image of SAOS2 xenografts. **C**, Tumor weight of SAOS2 tumors. p-values are from the Mann–Whitney test. **D**, Tumor-free survival of NSG mice bearing SAOS2 xenografts. p-values are from the log-rank (Mantel-Cox) test. **E**, IHC images showing Ki-67 and cleaved caspase 3 staining in SAOS2 xenografts. **F** and **G**, Quantitative analyses of Ki-67 (**F**) and cleaved caspase-3 (**G**)

Next, we tested whether JMJD1C is involved in the growth of OS xenograft. We designed two shRNAs to reduce the levels of JMJD1C in SAOS2 cells (Fig. 6A). Cells transduced with lentivirus expressing shLuc (luciferase shRNA as a control) or JMJD1C shRNAs were transplanted into the NSG mice. Tumors of JMJD1C shRNAs were significantly smaller than those of shLuc (Fig. 6B, C). Depletion of JMJD1C also prolonged the survival of host mice (Fig. 6D). IHC analyses showed that JMJD1C depletion phenocopied SOX9 depletion in terms of reduced Ki-67 staining and increased cleaved caspase 3 signal (Fig. 6E–G), suggesting that SOX9 and JMJD1C interact to promote OS growth.

## Discussion

**Fig. 7:**
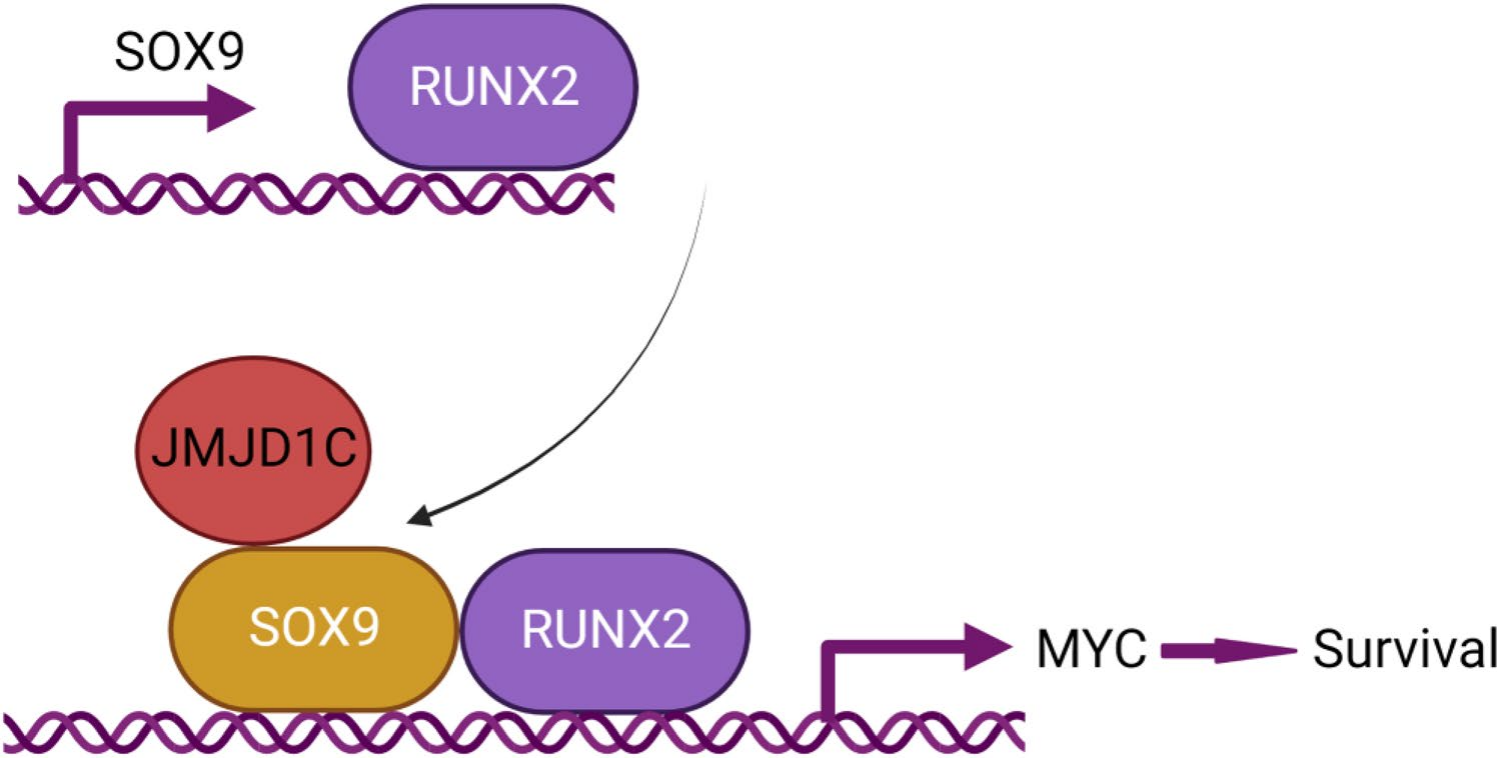
A model of RUNX2-SOX9-JMJD1C network in OS. RUNX2 induces *SOX9* transcription. SOX9 interacts with RUNX2 and JMJD1C to activate *MYC* to promote survival of OS cells

Transcriptional networks have been studied in several cancers, such as neuroblastoma and acute myeloid leukemia [27, 28]. However, the transcriptional circuit in OS remains largely unknown. In this study, we aimed to understand further the transcriptional network that is crucial for OS cell survival. We leveraged the knowledge that RUNX2 is a transcription factor dependency for OS [6] and discovered that SOX9 is an important component of the RUNX2 network (Fig. 7). Several lines of evidence support this conclusion. First, SOX9 is a downstream target of RUNX2. Second, SOX9 interacts with RUNX2 to induce the transcription of MYC, a known survival factor in OS and many types of cancer [6, 29]. Third, we identified JMJD1C as a novel interacting partner of SOX9. Last, JMJD1C knockdown and SOX9 knockdown phenocopy in the inhibition of OS tumor growth.

As to SOX9-regulated genes, we cautioned that it is unknown whether they are direct targets of SOX9 due to our unsuccessful attempts to perform ChIPseq of SOX9. This prevents us from further investigating the downstream targets that mediate SOX9's function. However, the regulation of MYC and enrichment of MYC-regulated genes strongly support the notion that the pro-survival function of SOX9 in OS is partially through MYC.

JMJD1C was originally discovered as a histone H3 lysine 9 methylation (mono/di) demethylase [30]. Recently, non-histone substrates, such as STAT3, emerged as JMJD1C's substrates [31]. Interestingly, STAT3 overexpression has been associated with poor prognosis of OS and the inhibition of JAK2/SATA3 by an inhibitor has shown promising result in OS tumor reduction in nude mice [32]. Although our study focues on SOX9, it is possible that JMJD1C regulating OS growth through affecting the activities of multiple TFs. It has been shown that JMJD1C plays a role in several types of cancer, such as leukemia [33]. In our study, we show that JMJD1C is critical for the growth of OS. Given that depletion of JMJD1C or SOX9 has similar effects on OS growth and these two proteins interact with each other, it is highly likely that JMJD1C may cooperate with SOX9 to drive OS cell survival. However, whether SOX9 recruits JMJD1C to demethylate histones or JMJD1C demethylates SOX9 or both remains unclear. Future studies should test these possibilities.

Our study has implications for the development of targeted therapies for OS. For example, the results from this study suggest that JMJD1C is an attractive target. Although there are currently no JMJD1C-specific inhibitors, once they become available, it will be interesting to test their effects on OS growth. The interaction between SOX9 and RUNX2 also raises the possibility of co-inhibiting the pathways of these two inhibitors. Previously, we found that RUNX2 recruits Menin to activate *MYC*. Mi-2 and Mi-3, two small-molecule inhibitors for Menin, impair RUNX2's function and increase apoptosis of OS cells [6]. Therefore, the co-inhibition of SOX9 and RUNX2 by combining JMJD1C-specific inhibitors with Mi-2 or Mi-3 may kill OS cells more effectively.

## Materials and methods

### Cell culture

Umbilical cord-derived (Cat#: PCS-500-010), adipose tissue-derived (Cat#: PCS-500-011), and bone marrow-derived (BM, Cat#: PCS-500-012) MSCs, SAOS2 (Cat#: HTB-85), U2OS (Cat#: HTB-96), HOS (Cat#: CRL-1543), HOS-MNNG (Cat#: CRL-1547), 143B (Cat#: CRL-8303), G292 (Cat#: CRL-1423), and MG63 (Cat#: CRL-1427) cells were purchased from ATCC and cultured per the vendor's instructions. Hu09-M112 cells were a kind gift from Jun Yokota (Biology Division, National Cancer Center Research Institute, Japan). DUNN cells were a kind gift from Dr. Chand Khanna and maintained in DMEM plus 10% FBS + 1% antibiotics [34]. SC2.LM cells were isolated from a spontaneous tumor derived from an in-house mouse strain (SP7-Cre;p53^fl/+^).

### Immunoblotting

Immunoblotting was performed as previously described [35]. Briefly, cells were lysed in whole-cell lysis buffer followed by 5-min sonication (30 s on and 30 s off) at 4 °C. Protein concentration was determined by the Bradford assay, and the same amount of proteins were resolved on 4–12% Bis–Tris NuPAGE Protein gels (Fisher Scientific) and transferred to nitrocellulose membranes (Bio-Rad). Antibodies used are: SOX9 (Millipore, Cat#: AB5535), cleaved caspase 3 (Cell Signaling, Cat#: 9664S), β-actin (Sigma, Cat#: A5316).

### Reporter assay

The DNA sequence containing the putative RUNX2 response element was cloned into pGL4.23[luc2/minP] (Promega, GenBank^®^ Accession Number DQ904455) by using oligos (5ʹ-CACCCTCGAGGAC-TGTATCTCCAAAAATCTAGG-3ʹ and 5ʹ-ATACAAGCTTCATATTAAAACCAGAT-AAGCAAG-3ʹ) and restriction enzymes (XhoI and HindIII) (Additional file 2: Figure S2). SAOS2 and 293 T cells were plated in 12-well plate at the density of 100,000 cells/well. The next day, cells were transfected with 300 ng of the reporter vector and 300 ng of an empty vector or a vector expressing RUNX2 plus 20 ng of pRL-SV40 (an internal control, Promega, Cat: E2231). Both firefly luciferase and *Renilla* luciferase activities were measured using Dual-Glo^®^ Luciferase Assay System (Promega, Cat: E2940). Normalized luciferase signal was calculated as firefly luciferase signal versus *Renilla* luciferase signal.

### Propidium Iodide (PI) staining

PI staining was carried out as previously described [6]. Cells were fixed and permeabilized using 70% ethanol at -20 degree overnight, washed 1X and resuspended with PBS. RNA was digested with 10 ug/ml DNase-free RNase for 1 h at room temperature. Propidum iodide (Sigma, Cat#: P4170)was added with a final concentration of 100 ug/ml before loading on a flow cytometer.

### Tumor growth

All animal procedures reported in this study that were performed by NCI-CCR affiliated staff were approved by the NCI Animal Care and Use Committee (ACUC) and in accordance with federal regulatory requirements and standards. All components of the intramural NIH ACU program are accredited by AAALAC International. One million SAOS2 cells transduced indicated shRNAs were transplanted into the hind limb muscle close to the femur. When a mouse in the experiment had a tumor larger than 2 cm in diameter, all mice were euthanized, and tumors were dissected for the downstream analyses.

### Immunohistochemistry

Formalin fixed paraffin embedded slides were deparaffinized in xylene, followed by 100% and 95% ethanol treatment. Antigens were retrieved by boiling slides in 10 mM sodium citrate for 10 min. After cooling, slides were treated with 3% H2O2 for 10 min to block endogenous peroxidase activity, followed by PBS + 0.1% Tween 20 washing and blocked with serum. Slides were then incubated with 1:100 Ki-67 antibody (Cell Signaling, Cat#: 12202S) or 1:50 cleaved caspase 3 antibody (Cell Signaling, Cat#: 9661S) for 1 h at room temperature, washed three times with PBS, incubated with biotinylated goat anti-rabbit IgG secondary antibody (VECTASTAIN ABC Kit) for 1 h at room temperature. After washing with biotin-avidin solution for 30 min at room temperature, slides were rinsed with PBS three times, DAB solution was added to allow color development for 2–5 min.

### BioID2

DUNN cells stably expressing retroviral expression plasmids, pBabe-BioID2-HA-puro (EV, Addgene, Plasmid #120308, a gift from Kyle Roux) and pBabe-SOX9-BioID2-HA-puro, were generated using a retroviral transduction system. Two million Platinum-A (Plat-A) cells were grown on a collagen-coated 60 mm dish in DMEM, 10% FBS media without any antibiotics. The next day, cells were transfected with 3 µg of the above-mentioned retroviral expression plasmids along with 150 µl of Opti-MEM and 9 µl of FuGENE transfection reagent (Promega, Cat# E2311). Retroviral supernatant was harvested 48 h after transfection. 500 µl of retroviral supernatant, 1500 µl of DMEM/F12 media, and 6 µg/ml polybrene were used to transduce DUNN cells. Subsequently, cells were transferred to media containing 2 µg/ml puromycin to generate stable cell lines expressing SOX9-BioID2-HA or the empty vector. For biotin pull-down, 8 million cells were grown in four 10-cm dishes, harvested, washed 2X with PBS, and lysed in 2 ml of lysis buffer (50 mM Tris pH 7.4, 500 mM NaCl, 0.4% SDS, 2% Triton X-100, 1 mM DTT, 5 mM EDTA, and protease inhibitors). Subsequently, cell lysates were sonicated for 5 min with 30 s ON and 30 s OFF using a Bioruptor sonicator. Next, the sonicated lysate was centrifuged at 13000 rpm and the supernatant was collected for protein estimation. 4 mg of protein from each sample was incubated with 150 µl of streptavidin magnetic beads (New England Biolabs, Cat# S1420S) overnight at 4 °C with mixing. The next day, beads were collected using a magnetic stand and washed twice with 2% SDS for 10 min, followed by 1X wash with 0.1% sodium deoxycholate, 1% Triton X-100, 500 mM NaCl, 1 mM EDTA, and 50 mM HEPES pH 7.5 for 10 min. Next, beads were washed 1X with 250 mM LiCl, 0.5% NP-40, 0.5% sodium deoxycholate, 1 mM EDTA and 10 mM Tris pH 8.0 for 10 min, followed by 2X wash with 50 mM Tris pH 7.4, 150 mM NaCl for 5 min each. Finally, beads were washed once with 25 mM HEPES pH 7.3 for 5 min and resuspended in 100 µl of 25 mM HEPES and sent to the NCI Protein Laboratory for mass spectrometry analysis**.** The total number of identified peptides (peptide spectrum matches) for a specific protein was used to calculate the enrichment.

### Proximity ligation assay (PLA)

PLA was performed using the Duolink^®^ In Situ Detection Reagents Red (Sigma, Cat#: DUO92008-100RXN). Briefly, 2.5 × 10^4^ cells were grown on a Millicell EZ slide (Sigma, Cat#PEZGS0816) for 48 h. For PLA labeling, cells were fixed with 4% paraformaldehyde in PBS for 15 min, permeabilized with 0.3% Triton X-100 for 10 min, and then blocked with Duolink blocking solution at 37 °C for 1 h. Samples were subsequently probed with the primary antibodies overnight at 4 °C. SOX9 antibody (Novus Biologicals, Cat#: H00006662-M02, 1:100), RUNX2 antibody (MBL, Cat#: D130-3, 1:100) and JMJD1C antibody (Bethyl, Cat#: A300-884A, 1:100) were used. Ligation and amplification of probes were performed per the manufacturer's instructions. Images were captured using a Zeiss LSM 880 confocal microscope.

### Microarray analyses

To assess the expression levels of JMJD1C in OS tumors and normal tissues, we extracted microarray data from the GEO database using series numbers GSE12865, GSE14359, GSE14827, GSE16088, GSE16091, and GSE73166, as described previously [11].

### RNAseq analyses

For RNAseq, RNA was extracted from cells using Trizol and quality-controlled by using the TapeStation (Agilent) to make sure that the RNA integrity number (RIN) was larger than 8. RNAseq was performed by the Next Generation Sequencing Core at the Center of Cancer Research, National Cancer Institute.

## Declataions

## Supplementary Information

**Additional file 1: Table S1**. RUNX2 direct targets in SAOS2 cells.

**Additional file 2: Figure S1.** Regulation of SOX9 by RUNX2 in mouse OS cells. CRISPR/Cas9 was used to cause a short-term reduction of RUNX2 in SC2.LM cells, as long-term reduction of RUNX2 led to cell death. Immunoblotting was used to study the effect of RUNX2 reduction on SOX9. **Figure S2.** DNA Sequence containing RUNX2 response element downstream of the SOX9 locus. This entire sequence corresponds to the RUNX2 ChIPseq peak in Figure 1E, which was cloned into the reporter plasmid and used in the reporter assays shown in Figure 1F. The sequence highlighted in red is the putative RUNX2 binding motif.

**Additional file 3: Table S2**. SOX9-regulated transcripts.

**Additional file 4: Table S3**. Proteins bound by SOX9.

**Additional file 5: Table S4.** Transcription factors bound by SOX9.

## Data Availability

The datasets used and materials in this study are available upon reasonable request.
