# Supplementary material for: SOX9 is a key component of RUNX2-regulated transcriptional circuitry in osteosarcoma
Source: Cell Biosci. 2023 Jul 25;13:136. doi: 10.1186/s13578-023-01088-2 (PMC10367263; doi:10.1186/s13578-023-01088-2)
Supplement: Supplementary file 2 — Additional file 2: Figure S1. Regulation of SOX9 by RUNX2 in mouse OS cells. CRISPR/Cas9 was used to cause a short-term reduction of RUNX2 in SC2.LM cells, as long-term reduction of RUNX2 led to cell death. Immunoblotting was used to study the effect of RUNX2 reduction on SOX9. Figure S2. DNA Sequence containing RUNX2 response element downstream of the SOX9 locus. This entire sequence corresponds to the RUNX2 ChIPseq peak in Figure 1E, which was cloned into the reporter plasmid and used in the reporter assays shown in Figure 1F. The sequence highlighted in red is the putative RUNX2 binding motif. [file 13578_2023_1088_MOESM2_ESM.pptx]

## Slide 1
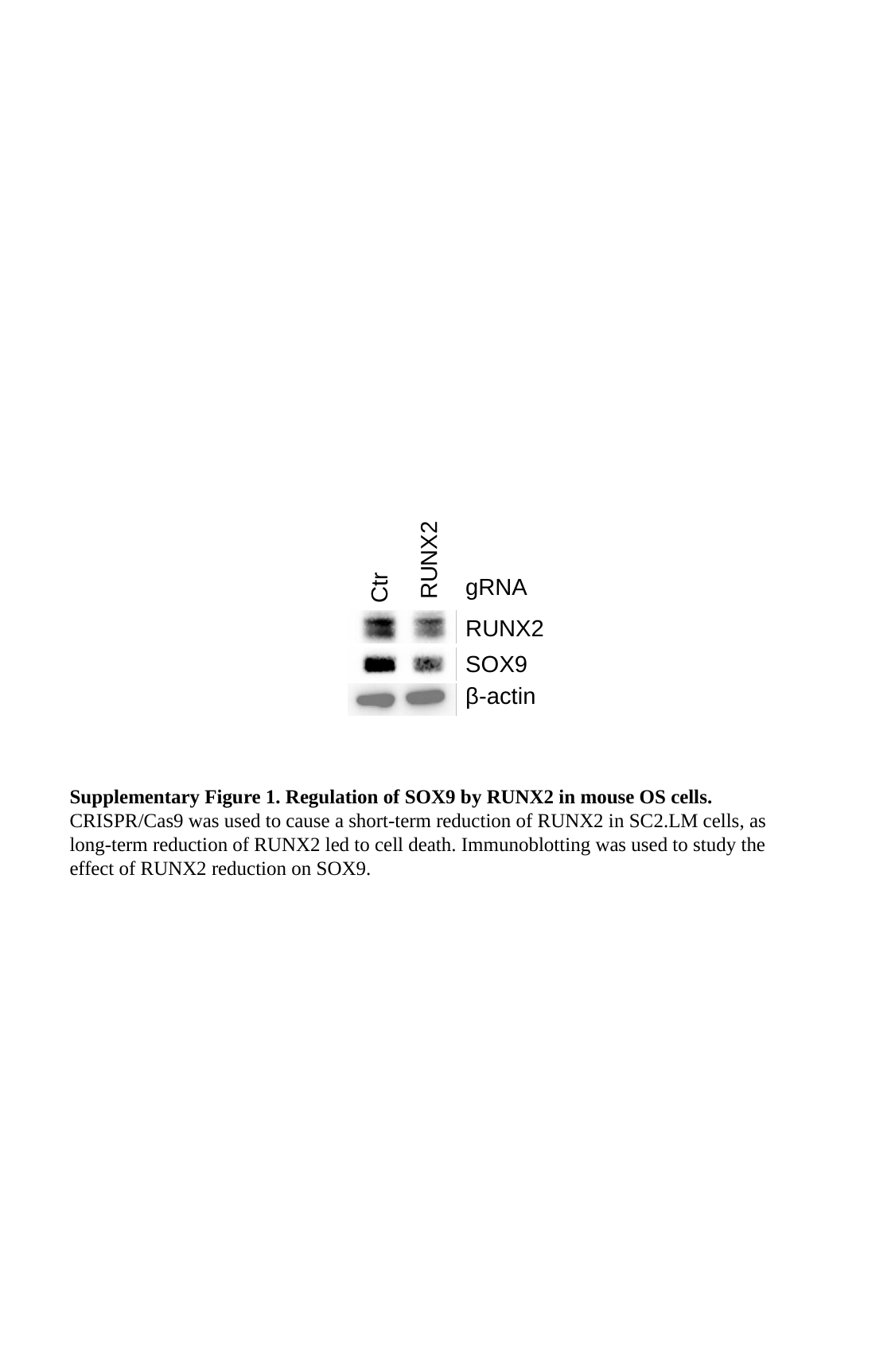

RUNX2
gRNA
Ctr
RUNX2
SOX9
β-actin
Supplementary Figure 1. Regulation of SOX9 by RUNX2 in mouse OS cells. CRISPR/Cas9 was used to cause a short-term reduction of RUNX2 in SC2.LM cells, as long-term reduction of RUNX2 led to cell death. Immunoblotting was used to study the effect of RUNX2 reduction on SOX9.

## Slide 2
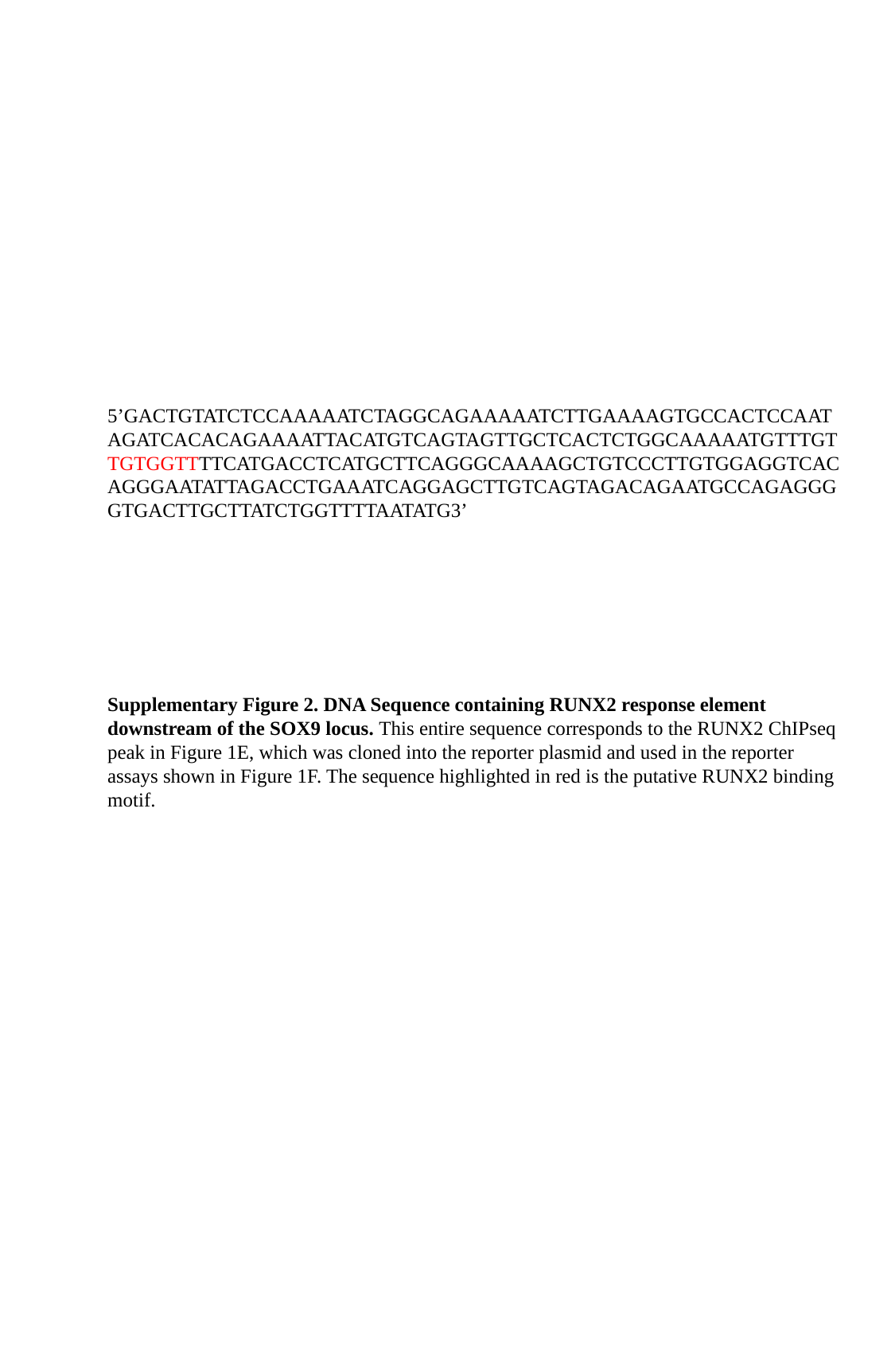

5’GACTGTATCTCCAAAAATCTAGGCAGAAAAATCTTGAAAAGTGCCACTCCAATAGATCACACAGAAAATTACATGTCAGTAGTTGCTCACTCTGGCAAAAATGTTTGTTGTGGTTTTCATGACCTCATGCTTCAGGGCAAAAGCTGTCCCTTGTGGAGGTCACAGGGAATATTAGACCTGAAATCAGGAGCTTGTCAGTAGACAGAATGCCAGAGGGGTGACTTGCTTATCTGGTTTTAATATG3’
Supplementary Figure 2. DNA Sequence containing RUNX2 response element downstream of the SOX9 locus. This entire sequence corresponds to the RUNX2 ChIPseq peak in Figure 1E, which was cloned into the reporter plasmid and used in the reporter assays shown in Figure 1F. The sequence highlighted in red is the putative RUNX2 binding motif.
